# Maternal diabetes causes developmental delay and death in early-somite mouse embryos

**DOI:** 10.1038/s41598-017-11696-x

**Published:** 2017-09-15

**Authors:** Jing Zhao, Theodorus B. M. Hakvoort, Jan M. Ruijter, Aldo Jongejan, Jan Koster, Sigrid M. A. Swagemakers, Aleksandar Sokolovic, Wouter H. Lamers

**Affiliations:** 1 Tytgat Institute for Liver and Intestinal Research, Academic Medical Center, Amsterdam, Netherlands; 2 Department of Anatomy, Embryology & Physiology, AMC, Amsterdam, Netherlands; 3 Bioinformatics Laboratory, Department of Clinical Epidemiology, Biostatistics & Bioinformatics, AMC, Amsterdam, Netherlands; 4 Department of Oncogenomics, AMC, Amsterdam, Netherlands; 5 Department of Informatics, Erasmus Medical Center, Rotterdam, Netherlands

## Abstract

Maternal diabetes causes congenital malformations and delays embryonic growth in the offspring. We investigated effects of maternal diabetes on mouse embryos during gastrulation and early organogenesis (ED7.5–11.5). Female mice were made diabetic with streptozotocin, treated with controlled-release insulin implants, and mated. Maternal blood glucose concentrations increased up to embryonic day (ED) 8.5. Maternal hyperglycemia induced severe growth retardation (approx.1 day) in 53% of the embryos on ED8.5, death in most of these embryos on ED9.5, and the termination of pregnancy on ED10.5 in litters with >20% dead embryos. Due to this selection, developmental delays and reduction in litter size were no longer observed thereafter in diabetic pregnancies. Male and female embryos were equally sensitive. High-throughput mRNA sequencing and pathway analysis of differentially expressed genes showed that retarded embryos failed to mount the adaptive suppression of gene expression that characterized non-retarded embryos (cell proliferation, cytoskeletal remodeling, oxidative phosphorylation). We conclude that failure of perigastrulation embryos of diabetic mothers to grow and survive is associated with their failure to shut down pathways that are strongly down-regulated in otherwise similar non-retarded embryos. Embryos that survive the early and generalized adverse effect of maternal diabetes, therefore, appear the subset in which malformations become manifest.

## Introduction

The incidence of diabetes is increasing annually world-wide^1^, including that in pregnant young adults^2^. Preexisting diabetes affects over 1% of all pregnancies and is rising in prevalence. Diabetic pregnancies are associated with embryonic growth delay^3^ and increase the risk for congenital malformations in the offspring^4–6^.

Rodent models of pregestational diabetes are usually based on alloxan- or streptozotocin (STZ)-induced diabetes in females. Neural-tube and cardiovascular malformations are often seen^7–9^, and may result from an adverse effect of high glucose concentrations on the normal development of neural crest cells^10,11^. Neural-tube defects range from a wavy neural tube via spina bifida at various locations to exencephaly and craniorhachischisis^12^, while cardiovascular malformations typically display defects of the outflow tract, atrial septum and atrioventricular valves, and extensive apoptosis^9,13^. The frequency of these malformations in several mouse strains is 10–15% of live ED10.5–13.5 embryos^7,9,13,14^. In rats^15^, but possibly not in mice^16^, pregnancies in such preconceptionally diabetic females are accompanied by growth retardation, developmental delay, embryonic death, and increased perfusion of the affected implantation sites^17^.

The rodent embryo is most sensitive to the teratogenic effect of maternal hyperglycemia if exposed during the pre- and early somite stages of development (ED7.5–8.5 in the mouse)^8,18,19^. Accordingly, mutant mouse models suggest that perturbations of mesoderm specification and cell migration during and shortly after gastrulation can cause neural-tube and cardiovascular defects^20^. We reported recently that maternal hyperglycemia impaired expression of genes involved in cell proliferation in non-retarded ED8.5 mouse embryos, and genes involved in cytoskeletal remodeling and oxidative phosphorylation in non-retarded mouse ED9.5 embryos^21^. A quarter of the differentially regulated genes (mostly down-regulated) were associated with neural-tube or cardiovascular defects if deficient^21^. Studies of older mouse embryos (ED ≥ 10.5) with manifest diabetic embryopathy showed that maternal hyperglycemia affected the expression of genes involved in apoptosis, proliferation, migration and differentiation during organogenesis in the offspring^9,12,22–24^. This change in the spectrum of affected genes is compatible with developmental stage-specific adaptation of gene expression.

A major difference between our and earlier reported findings is that many ED8.5 and ED9.5 mouse embryos of diabetic mothers (“experimental” embryos) were developmentally retarded, whereas growth retardation and developmental delay were explicitly reported as not being observed in ED10.5 litters of diabetic dams^23,25^. In the present study, therefore, we investigated the developmental progress of embryos from diabetic mothers between ED7.5 and ED11.5 to determine if the manifestation of diabetic embryopathy at and after ED10.5 was preceded by a more general adverse effect on embryonic development. The study was performed in the FVB mouse strain, which is susceptible to malformations induced by maternal hyperglycemia^26^. Diabetes was induced with streptozotocin and treated with insulin implants, so that severe hyperglycemia only developed after implantation of the embryos^7^. Pathway analysis after high-throughput mRNA sequencing of one-day retarded experimental embryos revealed that these embryos typically failed to mount the adaptive and generally suppressive response in gene expression that characterized the ED8.5 and ED9.5 experimental embryos that were not retarded.

## Results

### Biometric data of the mouse model of diabetic pregnancy

The details about the efficacy of the STZ treatment protocol can be found in our previous study^21^. All mice treated with STZ became diabetic. Blood glucose concentrations reached steady-state levels (approx. 25 mmol/L) within 3 weeks after inducing diabetes with STZ. After administering the controlled-release insulin implants, glucose levels declined but remained higher (approx. 14 mmol/L) in diabetic than in nondiabetic mice. Due to the fixed release rate of insulin and the increasing demand for insulin with progressing pregnancy^7^, blood glucose concentrations increased again 1.7-fold in the first week of pregnancy and plateaued after ED8.5. Only pregnant females with blood glucose concentrations exceeding 17 mmol/L at sacrifice were included in the analysis.

**Figure 1:**
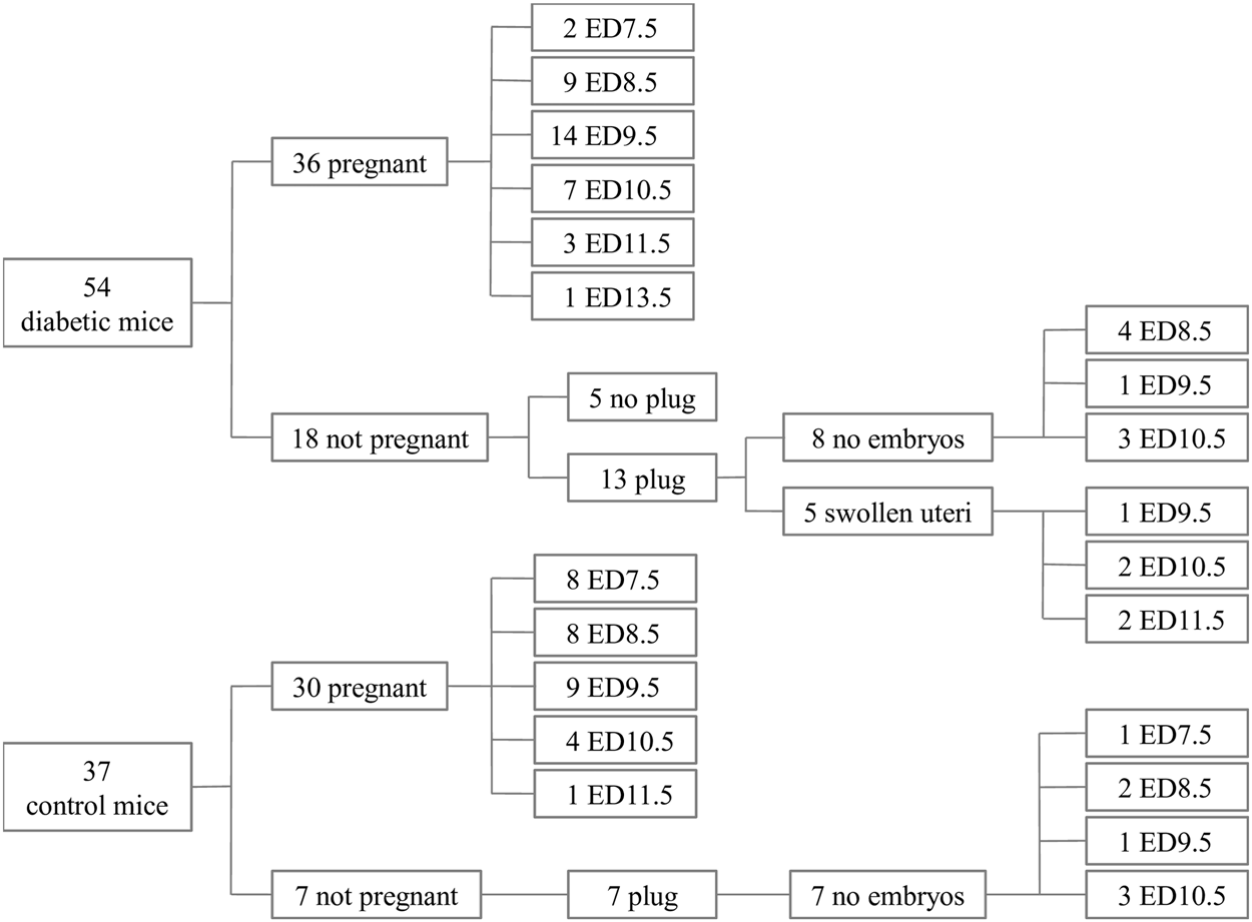
Fecundity of female diabetic and control mice and the gestational age at which the mice were killed. “Plug” and “no plug” indicate mice with or without visible vaginal plug. “No embryos” indicates that a vaginal plug was seen, but a normal uterus without embryos was found at harvest (copulation, but no conception), whereas a “swollen” uterus indicates that a vaginal plug was seen and a uterus without embryos that was swollen and reddish with engorged blood vessels was found at harvest. All EDs represent the embryonic day when the mice were sacrificed. The numbers indicate the number of mice sacrificed.

Figure 1 shows that among 54 female diabetic mice, 18 (33%) did not become pregnant within 3 weeks after being exposed to a proven fertile male. Of these 18 mice, a vaginal plug was observed in 13 females (72%). At sacrifice, 5 of the 13 uteri (38%) were swollen and congested with blood, as occurs after death of all embryos. In the non-diabetic control groups, 7 out of 37 mice (19%) did not become pregnant within 3 weeks (no difference with the STZ group, P = 0.13). No swollen and congested uterus was observed in any of these mice. The average litter size of control mice was 9.4 ± 0.5.

**Figure 2:**
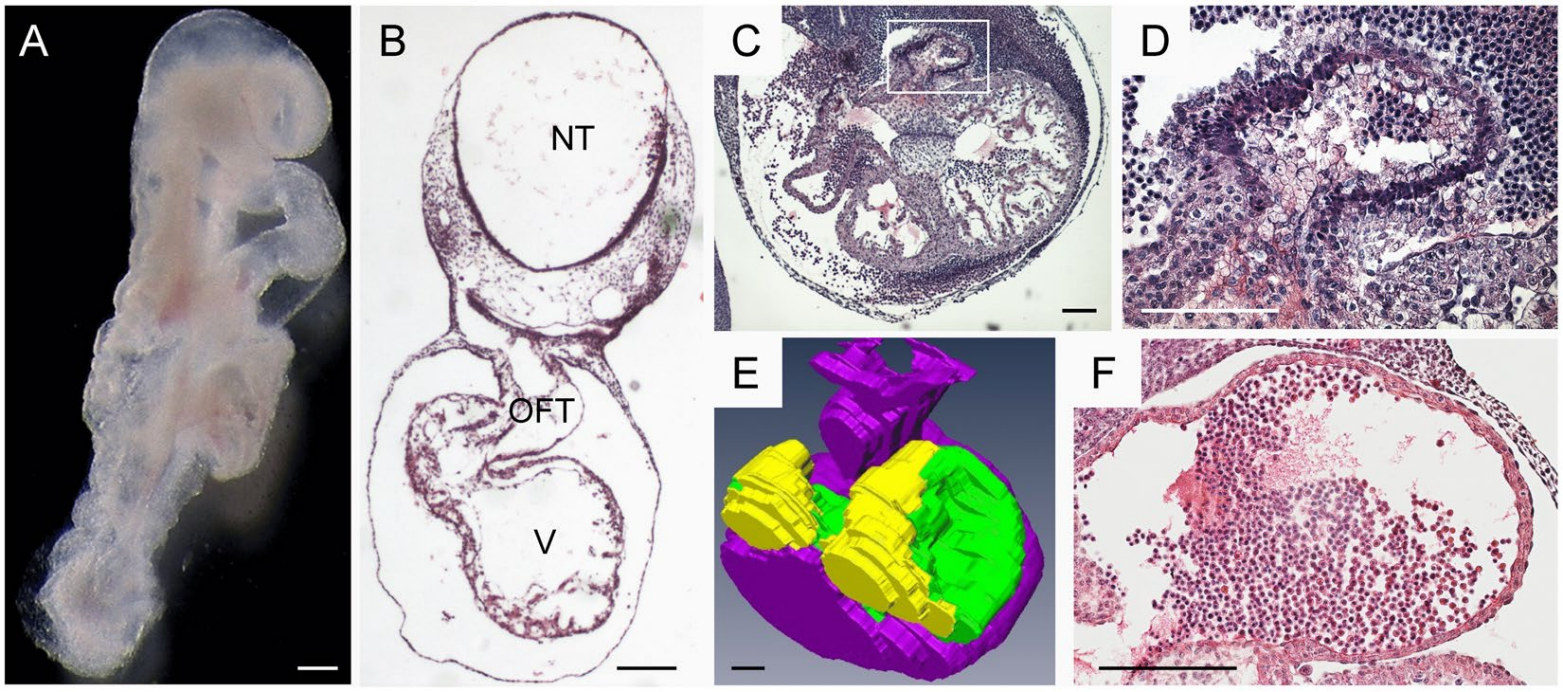
Growth retardation and apoptosis in embryos of diabetic mothers. Panel A shows a retarded experimental ED9.5 embryo with an incompletely turned caudal part of the body (“straight tail”) and a simple heart tube, which are compatible with a one-day delayed development. Panel B shows a hematoxylin and eosin-stained section of a delayed ED9.5 embryo. NT: dilated neural tube; OFT: outflow tract; V: ventricle, both in dilated pericardial cavity. Panels C and D (D is a magnification of the box in C) shows a hematoxylin and eosin-stained heart of an ED10.5 embryo from a diabetic mouse. Note the massive pycnotic fields in the dorsal wall of the embryonic atria. The 3D reconstruction (panel E; dorsal view of the heart region) identifies the pycnotic regions (yellow) in the left and right atria (green: the unaffected region; purple: ventricles and outflow tract). Panel F shows the hematoxylin and eosin-stained heart of a control ED10.5 embryo. Scale bars: 100 μm.

An inventory of the visible congenital malformations in 10 litters of ED9.5–11.5 embryos of pregnant diabetic mice yielded 12 abnormal embryos among in total 96 embryos (13%; Supplemental Table 1), which is similar to that found in literature^7,22^. Among the abnormal embryos, neural-tube defects (“wavy” neural tube and spina bifida, sometimes at several locations) were prevalent, as reported^12^. Some embryos were also retarded in development. Figure 2A shows, for example, an ED9.5 embryo with a simple tubular heart and a short, straight tail, features which are typical for embryos at ED8.5^27^. Additionally, the embryo in Fig. 2B has enlarged pericardial and neural-tube cavities, which is suggestive for cardiac failure. Other embryos had extensive fields of pycnotic nuclei in both embryonic atria (Fig. 2C–E) compared to control embryo (Fig. 2F). These morphological findings are consistent with those reported earlier in rat^15^ and mouse embryos^7,11,12,14^.

### Maternal hyperglycemia causes embryonic growth retardation on ED8.5 and death on ED9.5

Since growth retardation appeared a common feature in embryos with maternal hyperglycemia and since ED7-8 was reported as the most sensitive period for the teratogenic effect of hyperglycemia in mice^8^, we investigated the frequency of delayed development in ED7.5-10.5 embryos. Criteria used to stage embryos under the dissecting microscope were the number of somites that had developed, the “turning” of the embryo for ED8.5 embryos and the development of the head and heart for ED9.5 embryos^27^. Table 1 shows the effect of maternal hyperglycemia on embryonic growth. In the control group, growth retardation occurred in 5% and 8% of the embryos on ED8.5 and ED9.5, respectively, without sex difference. In the diabetic group, however, 53% of the embryos had incurred a severe growth retardation, equivalent to approx. 1 day, on ED8.5 (P < 1E-9 *vs* non-diabetic group). On ED8.5, 3 litters consisted of retarded embryos only, in 2 litters 60–70% of the embryos were retarded, and in 4 litters 10–40% of the embryos were retarded. Intriguingly, only 14% of the retrieved embryos with maternal hyperglycemia were retarded on ED9.5 (P < 1E-9 *vs* ED8.5; 9 litters without retarded embryos, 4 litters with 1–2 retarded embryos, and 1 litter with 6 retarded embryos) and still fewer (3%) on ED10.5 (2 embryos in 2 litters; Table 1). We reanalyzed the data shown in Fig. 1 of our previous report^21^ to see if there was any correlation between retarded embryonic growth and maternal blood glucose at mating, on ED7.5, ED8.5 or ED9.5, but found none (not shown).

**Table 1:** Effect of hyperglycemia on sex and embryonic growth.

treatment	ED	litters	male		female		sex not determined		pooled	
			non-retarded	retarded	non-retarded	retarded	non-retarded	retarded	non-retarded	retarded
Ctrl	7.5	7	22 (39%)	—	39 (52%)	—		7 (9%)	61	7
	8.5	7	28 (44%)	—	29 (46%)	—	3 (5%)	3 (5%)	60	3
	9.5	6	21 (40%)	—	23 (43%)	—	5 (9%)	4 (8%)	49	4
	10.5	4					36 (100%)	0	36	0
STZ	7.5	2					20 (100%)	0	20	0
	8.5	8	20 (25%)	21 (27%)	17 (21%)	21 (27%)	—	—	37*	42*
	9.5	12	27 (36%)	4 (5%)	35 (46%)	7 (9%)	3 (4%)	—	65	11
	10.5	7					66 (97%)	2 (3%)	66	2
Chi-squared overall P-value			ED8.5 and ED9.5 only						all EDs	
	ED		3.1E-05		1.10E-09		0.21		1.1E-13	
	treatment		4.0E-08		6.90E-09		0.16		3.8E-02	

FVB females were made diabetic and treated with insulin pellets. Embryos were recovered between ED7.5 and ED10.5, and considered alive if the heart was beating (after ED8.5) or if the RIN score of the isolated RNA was >9.0. “Non-retarded” indicates that the morphology of the embryos was as expected for gestational age, whereas “retarded” indicates the morphology of the embryo resembled the normal morphology one day earlier according to the criteria of Kaufman^27^. Embryos were processed for sex identification using PCR analysis. Numbers between brackets indicate percentage of male or female embryos. “Sex not determined” column indicates the number of embryos, of which gender identification failed (no PCR product; ED8.5 and ED9.5) or was not determined (ED7.5 and ED10.5). The statistical tests shown in the sex-separated groups were performed on the ED8.5 and 9.5 data only, whereas all embryonic ages were included in the pooled group. The frequencies of non-retarded and retarded embryos were compared with respect to embryonic day and treatment, using contingency-table statistics that resulted in overall Chi-squared significance. A within-Chi test identified treatment as the main source of retardation (P = 1.7E-12) and embryonic age as an additional condition (P = 0.0014). In the treated group the most significant changes occurred in embryos at ED8.5: a simultaneous reduction in number of normal and an increase in retarded embryos. *P = 1.3E-21.

**Figure 3:**
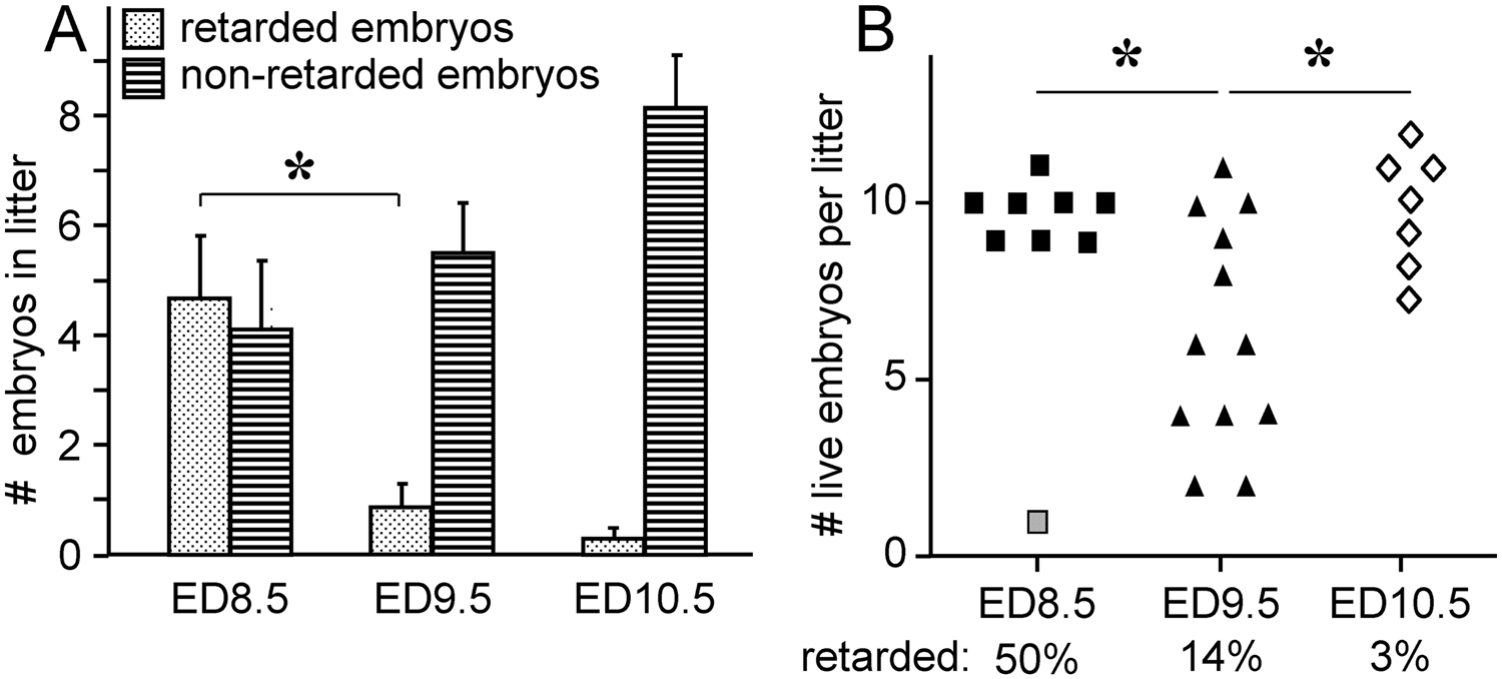
The effect of hyperglycemia on the prevalence of developmental retardation and litter size in pregnant diabetic mice. FVB females were made diabetic with streptozotocin and treated with insulin pellets. Embryos were harvested on ED8.5 (n = 9 litters), ED9.5 (n = 12 litters) and ED10.5 (n = 7 litters). Panel A: number of non-retarded and retarded embryos in a litter. “Non-retarded” indicates that the morphology of the embryos was appropriate for gestational age, whereas “retarded” indicates the morphology of the embryo resembled normal morphology one day earlier according to the criteria of Kaufman^27^. *P < 0.001. Panel B: number of live embryos in these diabetic litters. Gray symbol on ED8.5: one litter with only 1 embryo was considered an outlier (Grubb’s and Dixon’s outlier tests: P < 0.001 and <0.002, respectively). *P < 0.05. Note that approx. 50% of diabetic embryos were seriously retarded in development on ED8.5 (panel A), and that this was associated with a severe reduction in the number of live embryos per litter on ED9.5 (note “dumbbell” appearance of distribution) and the absence of these small litters on ED10.5 (panel B).

To track the fate of the retarded embryos, we determined the number of live embryos per litter for each age (Fig. 3 and Table 2). Maternal hyperglycemia resulted in growth retardation in 53% of ED8.5 embryos, but only few retarded ED9.5 or ED10.5 embryos were found (14% and 3%, respectively; Fig. 3A; P < 0.001). Coincident with the drop in the percentage of growth-retarded ED9.5 embryos in litters of diabetic mice, the number of live ED9.5 embryos decreased from 9.8 ± 0.3 on ED8.5 to 6.3 ± 0.9 on ED9.5 (Fig. 3B and Table 2; P = 0.021), indicating that many of the retarded embryos had died. More remarkably, the number of live embryos in litters of diabetic mice increased again to 9.7 ± 0.7 on ED10.5 (Fig. 3B and Table 2; P = 0.019). In conjunction with the apparent death of litters with a high percentage of retarded embryos (see previous section), we interpret the transient one-third reduction in the number of live embryos per litter in diabetic pregnancies on ED9.5 compared to ED8.5 and ED10.5 as showing that embryos that were severely growth-retarded on ED8.5 had died on ED9.5. We explain the apparent increase in the number of live embryos per litter on ED10.5 as a severe selection against and termination of litters of diabetic dams, in which >20% of the embryos were growth-retarded on ED8.5, because litters with few live embryos disappeared between ED9.5 and 10.5.

**Table 2:** The effect of hyperglycemia on litter size.

ED	# litters		litter size	
	Ctrl	STZ	Ctrl	STZ
7.5	7	2	10.7 ± 1.0	10, 10
8.5	7	8	9.0 ± 0.8	9.8 ± 0.3
9.5	6	12	8.8 ± 1.5	6.3 ± 0.9*
10.5	4	7	10.0 ± 0.9	9.7 ± 0.7

*Kruskal-Wallis test: P = 0.02 within STZ group.

FVB females were made diabetic and treated with insulin as described in the Materials and Methods section. Embryos were recovered between ED7.5 and ED10.5 and were considered alive if the heart was beating (after ED8.5) or if the RIN score of the isolated RNA was >9.0. The average number (±SEM) of embryos per litter is shown. The influence of developmental age and streptozotocin (STZ) treatment on litter size (SEM) was analyzed with the Kruskal-Wallis test.

### No sex difference in effect of hyperglycemia on embryonic growth

Because the sex of the embryo may determine its sensitivity to the teratogenic effects of maternal hyperglycemia^28^, we identified the male embryos by determining the presence of the *Sry* gene in the amniotic and chorionic membranes of ED7.5 and in the tails of older embryos. Table 1 shows that there was no significant difference in the percentage of male or female embryos that was growth-retarded on ED8.5 or ED9.5. In a small number of embryos, PCR amplification failed. Since this failure occurred as often in normal as in retarded embryos, it did not affect the interpretation of the data.

### Gene expression profile and pathway analysis of one-day delayed embryos

**Figure 4:**
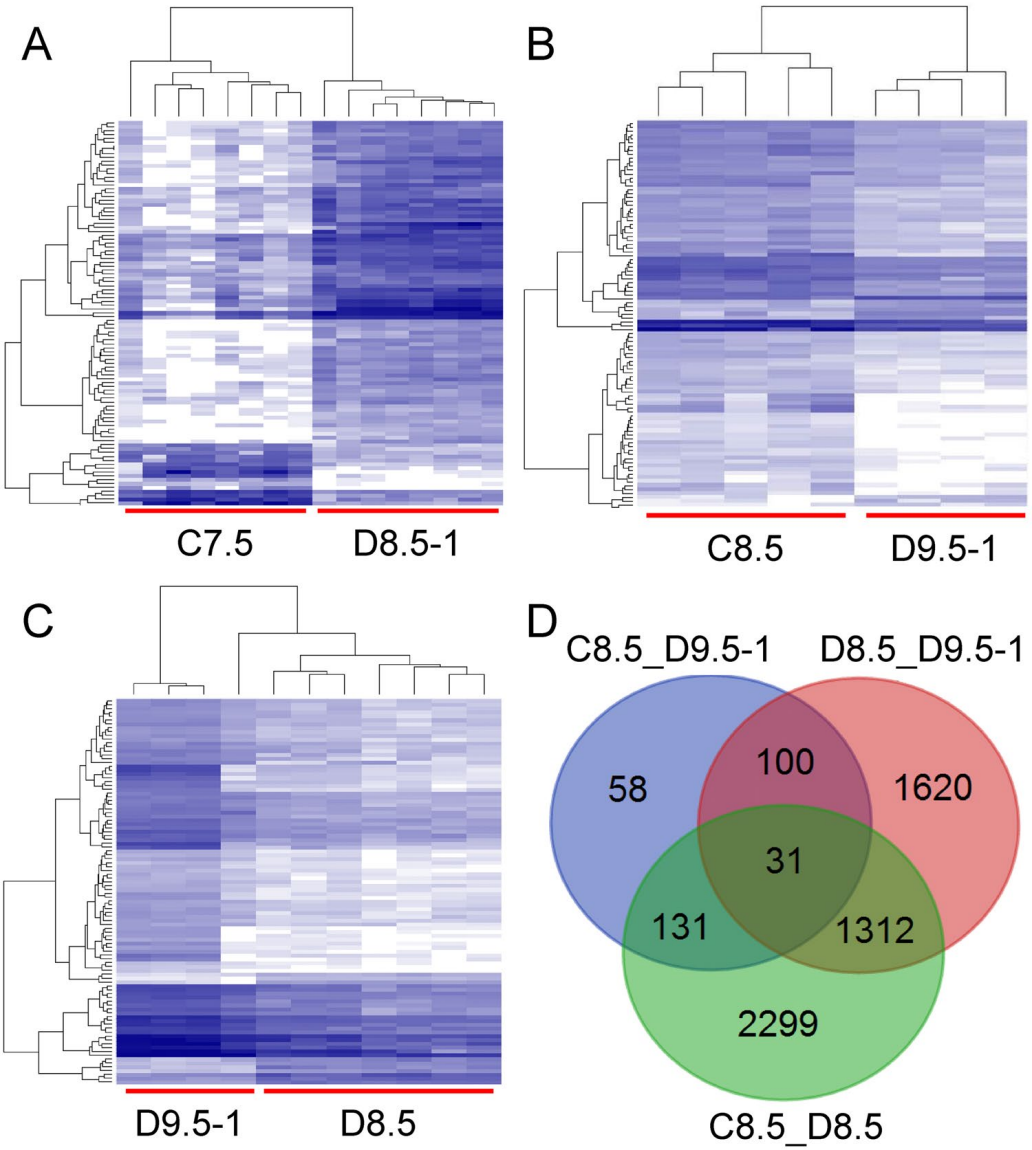
Hierarchical cluster analysis of gene expression in control and experimental one-day delayed ED8.5 and ED9.5 embryos. Panel A: Control ED7.5 embryos were compared to diabetic 1-day retarded ED8.5 embryos. Panel B: Control ED8.5 embryos to diabetic 1-day retarded ED9.5 embryos; Panel C: diabetic non-retarded ED8.5 embryos to diabetic 1-day retarded ED9.5 embryos. Hierarchical clustering was performed by GLM. The tree diagram on the top of the panels reflects the correspondence in gene expression between embryos, with less height of the branches indicating a higher similarity. The dendrograms at the left margin indicate the correlation in the response of the respective genes. The intensity of the blue color represents the relative level of expression of a gene (after transforming the data for variance stabilization). “C” and “D” at the bottom margin of the panels indicate “control” and “diabetic” embryos, respectively. Panel D: Venn diagram of the number of differentially expressed genes (without RIKEN, EST, unassigned and hypothetical gene sequences) in control and non-retarded diabetic ED8.5 embryos (C8.5_D8.5; green), in non-retarded and 1-day retarded diabetic embryos (D8.5_D9.5-1; red) and in control and 1-day retarded diabetic embryos (C8.5_D9.5-1; blue). The numbers in the overlapping areas show the number of genes differentially regulated in the two or three overlapping comparisons. Note that the number of differentially expressed genes is high if control embryos are compared with non-retarded diabetic embryos, but very small if control embryos are compared with retarded diabetic embryos.

Our previous study showed that maternal hyperglycemia mainly suppressed the expression of genes involved in cell proliferation in ED8.5 embryos and the remodeling of the cytoskeleton and oxidative phosphorylation in ED9.5 embryos^21^. To investigate the effects of growth retardation on gene expression, we performed SOLiD SAGE sequencing (accession number: PRJNA328571; study accession: SRP078567). In total, 8 ED7.5 embryos and 5 ED8.5 embryos from non-diabetic control mothers, and 7 ED8.5 embryos, 8 ED8.5 one-day delayed embryos (ED8.5-1), and 4 ED9.5 one-day delayed embryos (ED9.5-1) from diabetic mothers were screened. Clusters were generated with generalized linear models (GLM) based on the expression of the top 100 genes ranked by P-value, which separated the retarded from the non-retarded embryos in diabetic pregnancies and from the corresponding embryos in control pregnancies (Fig. 4A–C).

Differential gene expression was further analyzed with the Wald test in the DESeq2 package (available through Bioconductor)^29^. A P_adj_ < 0.05 was considered statistically significant. As summarized in Fig. 4D, 3773 genes were differentially expressed in experimental non-retarded compared to control ED8.5 embryos, whereas this number was only 320 if experimental retarded ED9.5-1 embryos were compared to control ED8.5 embryos. When non-retarded ED8.5 embryos were compared to ED9.5-1 embryos (both offspring of diabetic dams), 3063 genes were differentially expressed (Fig. 4D). These data show that gene expression in retarded experimental embryos was more similar to that in control embryos of the same developmental stage than to that in non-retarded experimental embryos.

**Figure 5:**
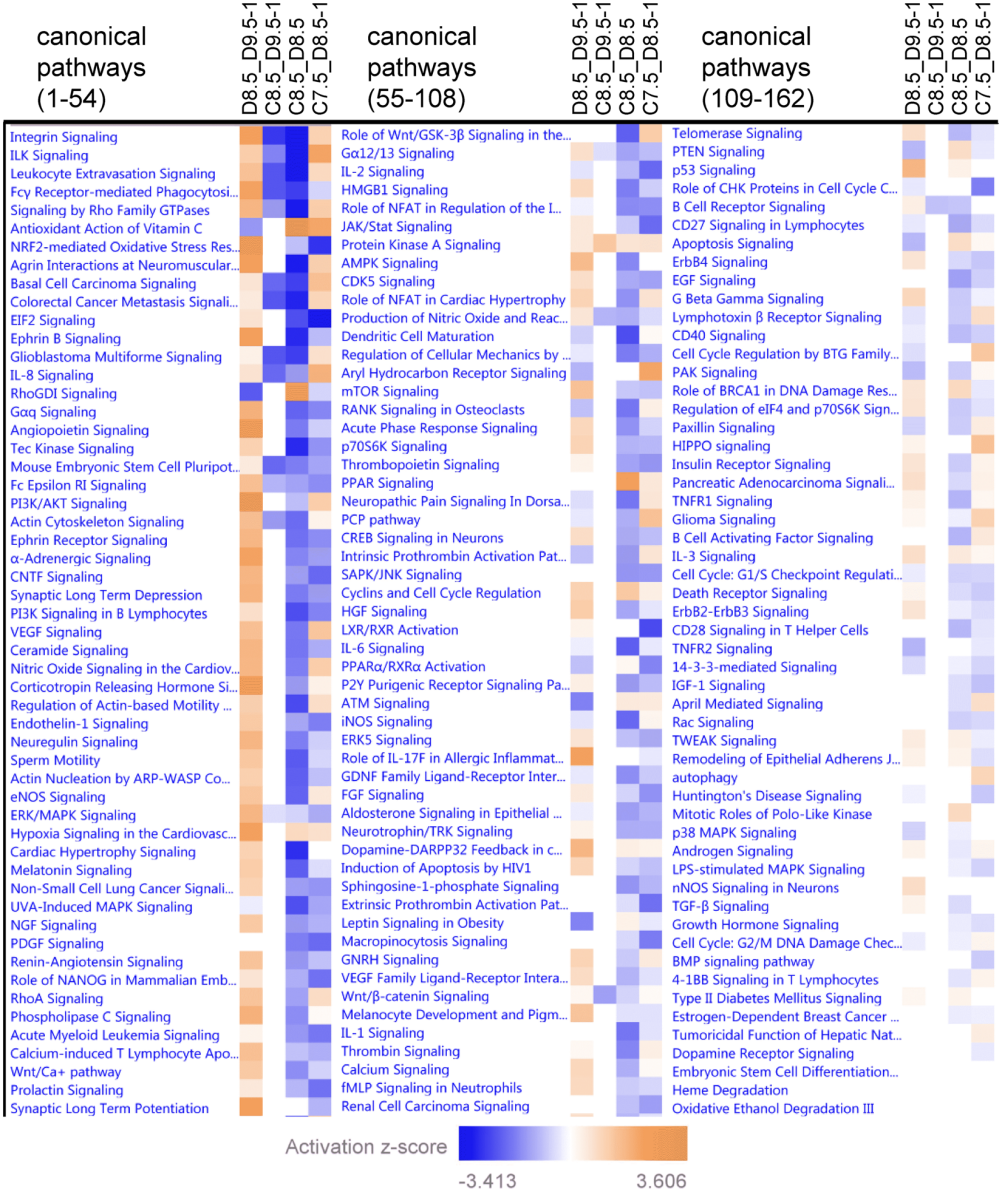
Pathway analysis of differences in gene expression between control and retarded or non-retarded experimental embryos. Non-retarded ED8.5 experimental embryos were compared to 1-day retarded ED9.5 experimental embryos (D8.5_D9.5-1; left column), ED8.5 control embryos to 1-day retarded ED9.5 experimental embryos (C8.5_D9.5-1; 2^nd^ column), ED8.5 control embryos to non-retarded ED8.5 experimental embryos (C8.5_D8.5; 3^rd^ column), and ED7.5 control embryos to 1-day retarded ED8.5 experimental embryos (C7.5_D8.5-1; 4^th^ column). Color intensity indicates stronger control, with blue and orange reflecting a negative and a positive Z-score, respectively, relative to the second term in the comparison. Note marked difference between 2^nd^ and 3^rd^ columns, which reflects the effect of growth retardation.

To find out whether the discrepancy between retarded and non-retarded embryos of diabetic dams converged on only a few metabolic or signaling pathways or, alternatively, indicated a failure of the retarded embryos to adapt to the maternal diabetic condition we performed pathway analysis of the differentially expressed genes. Figure 5 shows that, compared to control ED8.5 embryos, Integrin, Integrin-linked kinase (ILK), Rho-family GTPases and Ephrin-B signaling were most highly (down-)regulated in experimental ED8.5 embryos (see 3^rd^ column). Only genes involved in antioxidant action of vitamin C were significantly upregulated. These pathways concur largely with those we identified earlier^21^ on the MetaCore^TM^ platform (GeneGo, Inc., St. Joseph, MI, USA; for a list of the embryos compared in our previous^21^ and present study, see Supplemental Table 2). Relative to control ED8.5 embryos, experimental ED9.5-1 embryos differed from experimental ED8.5 embryos in a similar, but much weaker downregulation of pathways, so that only few of these pathways were significantly regulated (compare 2^nd^ and 3^rd^ columns in Fig. 5). This weaker downregulation of gene expression is also seen in the direct comparison of experimental ED8.5 and experimental D9.5-1 embryos (reversed color in 1^st^ column). The response in experimental ED8.5-1 of diabetic mothers compared to control ED7.5 embryos was more mixed: genes involved in ILK signaling and antioxidant action of vitamin C were e.g. highly upregulated, whereas NRF2-mediated oxidative stress and EIF2-mediated translation was significantly downregulated (4^th^ column). Unfortunately, we do not have expression profiles of non-retarded experimental ED7.5 embryos. These data suggest that growth-retarded embryos in diabetic pregnancies fail to shut down many pathways that are strongly down-regulated in otherwise comparable non-retarded embryos and that this failure leads to their demise between ED8.5 and ED9.5.

## Discussion

**Figure 6:**
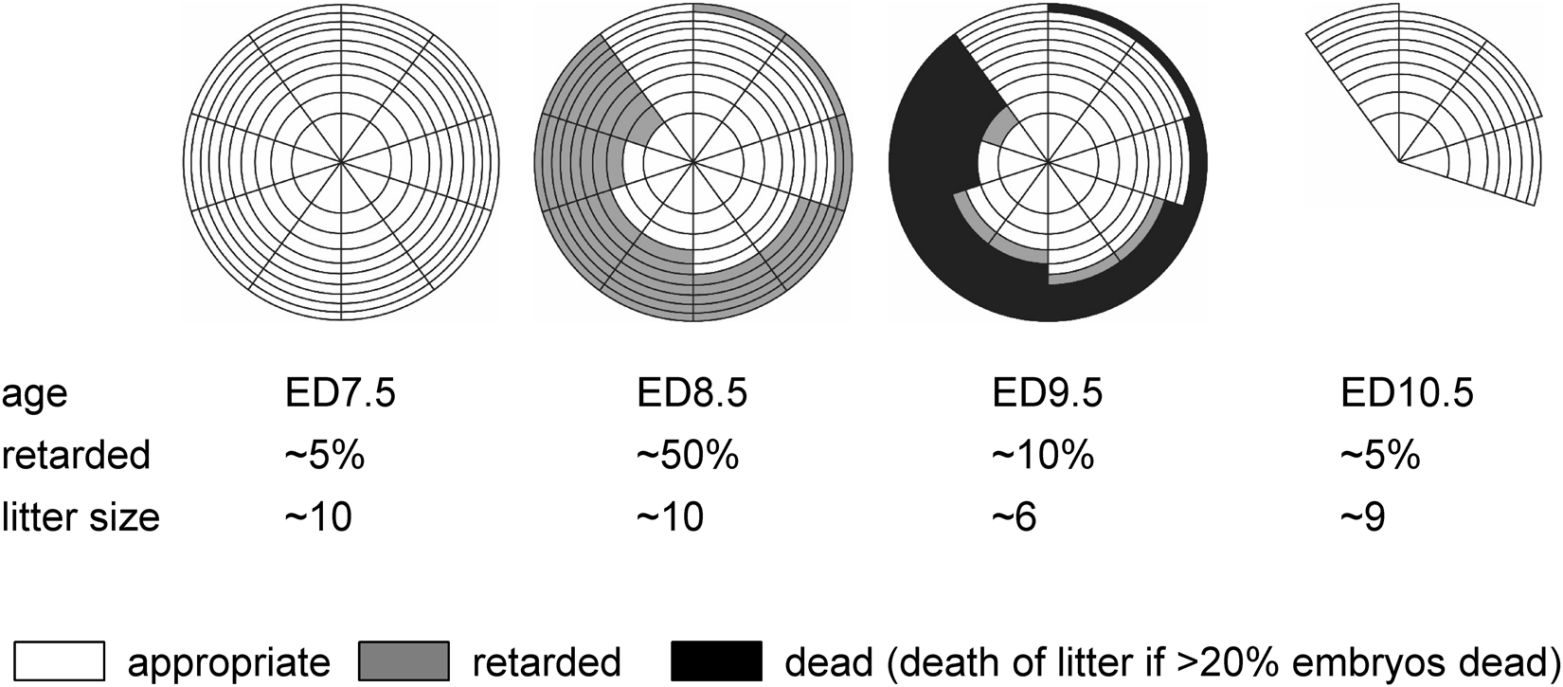
The fate of embryos in litters from diabetic mothers. Schematic representation of the fate of embryos in diabetic dams. This scheme, which is based on the data presented in Table 2 and Fig. 3, depicts the fate of 10 imaginary litters, with 10 embryos each, in the uterus of diabetic dams. Between ED7.5 and ED10.5, experimental embryos develop normally for gestational age (white), become delayed in development (gray), or die (black). The developmental delay in diabetic pregnancies typically developed between ED7.5 and ED8.5, and most embryos with a developmental delay died between ED8.5 and ED9.5. If >20% of the embryos in a litter were affected, the remaining embryos died between ED9.5 and ED10.5. Due to the demise of retarded embryos, average litter size declined from 10 on ED7.5 to 6 on ED9.5. As a result of selection against litters in which >20% of the embryos had died, small litters were no longer seen on ED10.5 and average litter size had increased again. The only trace of the affected litters was a lower percentage of pregnancies among diabetic relative to control females. The background rate of retarded development was approx. 5%.

In our experimental model, mouse embryos become sensitive to maternal hyperglycemia on ED7.5, as previously reported^8^, and develop visible malformations from ED10.5 onwards. Unexpectedly, we observed severe growth retardation in approx. 50% of the embryos of diabetic dams at ED8.5 and a approx. 30% decrease in litter size of these diabetic dams on ED9.5. Because we also observed diabetic dams with only dead embryos in their uterus, we interpret (for a graphical illustration of our reasoning, see Fig. 6) the transient decrease in litter size of diabetic dams on ED9.5 as showing that embryos that were severely growth-retarded on ED8.5, had died. The apparent normalization of the litter size of diabetic dams on ED10.5 can only be understood if diabetic pregnancies, in which >20% of the embryos had died from the sequels of maternal hyperglycemia, had terminated. As a result of this selection, no evidence (the percentage retarded embryos was not different from that in control pregnancies) of the deleterious effect of maternal hyperglycemia on embryonic growth was still demonstrable on ED10.5. Growth retardation was reported in ED11.5 rat embryos (26–29-somites, equivalent to ED10 mouse embryos^30,31^) when cultured in very high-glucose media^15,32^. Growth retardation and a temporary reduction in litter size were, however, not noted in diabetic mice^16^. However, in another study, a 0.5 day developmental delay on ED8.5 and a temporary reduction in litter size of diabetic pregnancies on ED9.5 were found, but the number of pregnancies studied was deemed too small to permit a conclusion^33^. These findings imply an early, rather general and possibly metabolic effect of maternal hyperglycemia during and immediately after gastrulation that leaves few traces due to the elimination of affected litters between ED9.5 and ED10.5, and a later, teratological effect that has thus far received most interest. If our finding can be extended to the human situation, the early effect would occur in the third week of gestation, so that the consequence, embryonic death, might not even be noted, except possibly for a perceived difficulty to become pregnant.

Even a temporary increase in circulating glucose concentration on ED7-8, that is, during gastrulation, suffices to bring about neural tube defects^8^. Our data shows that blood glucose levels in diabetic dams had increased to a plateau by that time^21^. Euglycemia during gastrulation is apparently crucial for normal embryonic development and further organogenesis (cf ref.^20^), because nearly half of the embryos of diabetic mice had developed a growth delay on ED8.5. Glycolysis is still the prevailing energy source in ED9.5 mouse embryos^21,34^, although studies in rat^35,36^ and mouse embryos^37^ suggest that the production of lactate decreases and mitochondrial activity slowly increases during early organogenesis. We observed that glycolytic activity and capacity had declined substantially in ED9.5 mouse embryos from diabetic mothers, suggesting that embryos of diabetic mothers had an energy problem^21^. In agreement, exposure of such embryos to a supernormal glucose concentration did not increase glucose oxidation^36,37^, but caused swelling of mitochondria^38^ and a significant decrease in reduced glutathione^39^.

These findings raise the question how such a general disturbance of metabolism can result in malformations in a restricted range of organ systems, such as the neural tube and heart. Our present data may explain this paradox, because shortly after becoming sensitive to hyperglycemia, embryos do suffer from a generalized adverse effect. Because embryos that were most severely affected by maternal hyperglycemia did not survive, the embryos with malformations appear to be those that were only relatively mildly affected by the early toxic effect of maternal hyperglycemia. The difference between the 2 groups of embryos appears to reside in their adaptive response in gene expression: the growth-retarded embryos were unable to adapt to the maternal diabetic condition by a pronounced, generally downward change in gene expression that was found in the non-retarded ED8.5 embryos ^21^. It is tempting to speculate that this failure to adapt to maternal hyperglycemia represents or includes a causal factor for the failure to grow and survive. The differential sensitivity of the (mouse) embryos to glucose may develop if the complex downstream effects of maternal hyperglycemia, including glutathione synthesis and oxidation, hexosamine synthesis, p53 upregulation and apoptosis^7,9,40,41^, are not strictly deterministic, but vary stochastically, so that the outcome is not uniform.

Non-retarded ED8.5 embryos of diabetic dams suffer mainly from impaired cell proliferation, and ED9.5 embryos from impaired cytoskeletal remodeling and oxidative phosphorylation^21^. Pathway analysis on one-day delayed ED8.5 experimental embryos revealed a remarkably muted adaptive response in gene expression relative to the non-retarded experimental embryos. Integrin (ILK) signaling, antioxidant action of vitamin C and NRF2-mediated oxidative stress remained most strongly affected in growth-retarded embryos. ILK is the major regulator of Integrin-mediated signaling. Integrins are cell-adhesion molecules that bind to the extracellular matrix (ECM) and forge a connection between the ECM and the actin cytoskeleton^42^. Oxidative stress emerges as a more prominent factor in the present analysis on the Ingenuity platform than our previous analysis on the MetaCore^TM^ platform, but also this response was suppressed in the growth-retarded mouse embryos.

## Conclusions

Our study showed that maternal hyperglycemia has an early and generalized adverse effect on embryonic development between ED7.5 and ED9.5 (equivalent to 18–28 days development in humans), whereas the characteristic malformations of the neural tube and cardiac outflow tract become apparent only thereafter. Hyperglycemia-mediated changes in gene expression were markedly absent in growth-retarded ED8.5 (mouse) embryos, while malformations developed in non-retarded, surviving embryos that were able to mount an adaptive response to hyperglycemia on ED8.5.

## Materials and Methods

### Animals

FVB mice (9–11 weeks old) were obtained from Harlan Sprague Dawley (Venray, The Netherlands) and fed an irradiated diet that was based on the Purina 9 F diet (http://labdiet.com/pdf/5020.pdf; production: ABDiets, Woerden, The Netherlands). Mice were kept on a 12-h light/12-h dark cycle at 22 °C with free access to water and food. The study was carried out in accordance with the Dutch Guidelines for the Care and Use of Laboratory Animals and approved by the Ethical Committee for Animal Research of the University of Amsterdam (ALC101225).

### Induction of diabetes

Diabetes was induced in female FVB mice with streptozotocin as recommended by the Animal Models of Diabetic Complications Consortium (AMDCC)^43^; and as described in our previous study^21^. In brief, animals to be treated with streptozotocin were randomly chosen. The diabetic group included 54 and the control group 37 mice. 4–5 diabetic or control mice were housed in one cage. Sixty mg/kg streptozotocin (STZ; Sigma, Zwijndrecht, The Netherlands), freshly dissolved in sodium citrate (0.1 M, pH 4.5), was injected intraperitoneally for 5 consecutive days. Hyperglycemia was treated with subcutaneous sustained-release insulin implants (LinSHIN, Toronto, Canada) that released approx. 0.1 U/day per implant for at least 30 days. Mice were mated to proven fertile males. Pregnant mice were sacrificed by cervical dislocation and embryos recovered on ED7.5, ED8.5, ED9.5, ED10.5, ED11.5, or ED13.5 (one litter only). Embryos were considered alive when their heart was beating (>ED8.5) or if the RIN number of RNA isolated from them was >9.0. The age of the embryos was verified by comparison with the criteria of Kaufman^27^.

### Sex identification

Because male human embryos were reported to be more sensitive to the diabetic state of their mothers than female embryos^44^, whereas the reverse was described for mouse embryos^45^, we studied male and female embryos separately. DNA from the visceral yolk sacs of ED8.5 embryos, or the posterior part (cut made just caudal to the heart) of ED9.5 embryos was isolated with TriPure Isolation Reagent (Roche, Almere, The Netherlands). The tissues were taken from the remaining parts of the embryos after harvesting the anterior part (ED8.5) and heart-containing segment (ED9.5) for mRNA quantification. The sex of the embryos was determined by a multiplex PCR amplification using primers to amplify the male-specific *Sry* gene and the autosomal *Il3* gene for reference^46^.

### Embryo morphology

ED8.5-ED10.5 embryos were fixed in Bouin’s fixative (saturated picric acid (15), formaldehyde (5), glacial acetic acid (1; v/v)) for 2 hours at 4 °C, stored in 70% ethanol, and embedded in paraffin. Serial transversely cut 7 μm-thick sections were stained with hematoxylin and eosin (HE), and photographed with a Leica DMRA2 microscope equipped with a Leica DM300 photo camera. Digital images from serial sections of mouse embryos were used to prepare three-dimensional reconstructions with Amira 5.2 software package (Visage Imaging, San Diego, CA, USA).

### Quantification of mRNAs by SOLiD SAGE sequencing

In our previous study, we compared gene expression in non-retarded control and experimental ED8.5 or 9.5 embryos^21^. In the present study, we compared gene expression in control ED7.5 and ED8.5 embryos with one-day delayed ED8.5 and ED9.5 experimental embryos, respectively. Total RNA was extracted from whole ED7.5 and one-day delayed ED8.5 embryos, and from the anterior half of embryos (transverse cut made just caudal to the heart) control ED8.5 and one-day delayed experimental ED9.5 embryos with the TriPure Isolation Reagent (Roche, Almere, The Netherlands). Embryos were frozen and thawed 3 times using liquid nitrogen. After centrifugation, the aqueous phase of the TriPure extract was re-extracted 3 times with choloroform:isoamyl alcohol (24:1) and precipitated with ethanol after addition of 10 µg glycogen. The quantity of total RNA was determined with a Qubit 1.0 Fluorometer (Invitrogen, The Netherlands), while the quality was assessed with a Bioanalyser RNA 6000 Nano Chip (Agilent Technologies, Amstelveen, The Netherlands). The RNA integrity number (RIN) of all samples was between 9.0 and 10.0. The total RNA yield per embryo was 1.0 ± 0.1 µg for one-day delayed ED8.5 embryos and 0.9 ± 0.1 µg for one-day delayed ED9.5 embryos. The relative abundance of individual transcripts was determined by the SOLiD SAGE approach, as in our previous study^21^. Pathway analysis of the differentially expressed genes was performed using QIAGEN’s Ingenuity Pathway Analysis (IPA®, QIAGEN Redwood City, www.qiagen.com/ingenuity).

### Statistical analyses

Blood glucose concentrations and litter size are expressed as mean ± SEM. Statistical analyses were performed using nonparametric Mann-Whitney U and Kruskal-Wallis tests. The number of normal and retarded embryos was compared with embryonic age and streptozotocin treatment as variables, using contingency-table statistics. Expected values were based on column and row totals or Mendelian expectation (gender). In case of a significant overall Chi-squared value, the contributions to the Chi-squared value were tested to determine the source of the observed difference. A P-value < 0.05 was considered statistically significant.

### Data availability statement

The datasets generated during and/or analysed during the current study are available in NCBI (accession number: PRJNA328571; SRA study: SRP078567). The datasets generated in our earlier study^21^ are also available in NCBI (accession number: PRJNA275285; SRA study: SRP056150).

## Electronic supplementary material

Supplemental Table 1 and 2
