# Supplementary material for: Maternal diabetes causes developmental delay and death in early-somite mouse embryos
Source: Sci Rep. 2017 Sep 15;7:11714. doi: 10.1038/s41598-017-11696-x (PMC5601907; doi:10.1038/s41598-017-11696-x)
Supplement: Supplementary file 1 — Supplemental Table 1 and 2 [file 41598_2017_11696_MOESM1_ESM.pdf]

Maternal diabetes causes developmental delay and death in early-somite mouse embryos

Jing Zhao[1], Theodorus B.M. Hakvoort[1], Jan M. Ruijter[2], Aldo Jongejan[3], Jan Koster[4], Sigrid M. A. Swagemakers[5], Aleksandar Sokolovic[1], and Wouter H. Lamers[1*]

[1]Tytgat Institute for Liver and Intestinal Research, Academic Medical Center, Amsterdam;

[2]Department of Anatomy, Embryology & Physiology, AMC, Amsterdam;

[3]Bioinformatics Laboratory, Department of Clinical Epidemiology, Biostatistics & Bioinformatics, AMC, Amsterdam

[4]Department of Oncogenomics, AMC, Amsterdam

[5]Department of Informatics, Erasmus Medical Center, Rotterdam

Corresponding author:

Wouter H. Lamers, Tytgat Institute for Liver and Intestinal Research, Academic Medical Center, Meibergdreef 69-71, 1105 BK Amsterdam, The Netherlands. Phone: +31-205665948; Fax: +31-205669190 ; E-mail: w.h.lamers@amc.uva.nl

Supplemental Table 1. Malformations in the offspring of 10 consecutive pregnant diabetic mice

litter	# embryos	resorbed or dead	abnormal	age (ED)	malformation
1	9	0	3	9.5	open neural tube (tail); retarded embryo; pycnotic cells in atrium
2	4	0	1	9.5	retarded embryo
3	12	0	1	10.5	pycnotic cells in atrium and open neural tube (body)
4	9	2	1	10.5	pycnotic cells in atrium and big pericardium
5	9	1	2	10.5	open neural tube; retarded heart development
6	11	0	0	10.5	none
7	10	0	3	10.5	dilated heart and retarded brain development; spina bifida; retarded heart development
8	11	0	0	10.5	none
9	11	2	1	10.5	spina bifida
10	10	4	0	11.5	none
total	96	9	12		

Supplemental Table 2. Comparisons of gene expression data made in Zhao et al (2016) and present study

Embryonic Day (ED)	treatment	Zhao, 2016	present
ED7.5	control		+ (8)
ED8.5	control	+ (5)	+ (5)
ED9.5	control	+ (8)	
ED8.5	diabetic	+ (7)	+ (7)
ED9.5	diabetic	+ (8)	
ED8.5-1	diabetic		+ (8)
ED9.5-1	diabetic		+ (4)
